# Assessment of the kidneys: magnetic resonance angiography, perfusion and diffusion

**DOI:** 10.1186/1532-429X-13-70

**Published:** 2011-11-15

**Authors:** Ulrike I Attenberger, John N Morelli, Stefan O Schoenberg, Henrik J Michaely

**Affiliations:** 1 Institute of Clinical Radiology and Nuclear Medicine, University Medical Center Mannheim, Heidelberg University, Mannheim, Germany; 2 Scott and White Memorial Hospital and Clinic - Texas A&M University Health Sciences Center, Temple, TX, USA

## Abstract

Renal magnetic resonance (MR) imaging has undergone major improvements in the past several years. This review focuses on the technical basics and clinical applications of MR angiography (MRA) with the goal of enabling readers to acquire high-resolution, high quality renal artery MRA. The current role of contrast agents and their safe use in patients with renal impairment is discussed. In addition, an overview of promising techniques on the horizon for renal MR is provided. The clinical value and specific applications of renal MR are critically discussed.

## Introduction

Since its clinical introduction in the 1990's magnetic resonance angiography (MRA) has become an increasingly important tool in the evaluation of the renal vasculature [1-3]. Its benefits compared to the clinical gold-standard method, digital subtraction angiography (DSA), are well-known and widely discussed: a lack of ionizing radiation, non-invasiveness, and no reliance on iodinated contrast agents, the latter being particularly important in patients with impaired renal function. Even at 1.5 T, renal artery MRA allows accurate detection of renal vascular disease such as fibromuscular dysplasia, arterial dissection, and venous thrombosis, in addition to allowing accurate quantification of vascular luminal narrowing in processes like renal artery stenosis [4]. The ongoing incorporation of higher field strength imaging into routine clinical practice has further improved this method. The signal-to-noise ratio (SNR) gains at 3 T compared to 1.5 T allow for utilization of parallel imaging acceleration (PI) factors up to 3 without a significant loss in image quality. Higher PI factors are potentially advantageous in several ways and can be utilized to increase spatial resolution or decrease acquisition times. Reductions in acquisition time serve to also reduce the potential for image degradation from diaphragmatic motion thus allowing for improved depiction of segmental renal arteries. The use of newer contrast agents, such as high relaxivity or protein binding Gadolinium (Gd)-chelates, also improves image quality, particularly when combined with SNR gains available at 3 T. These factors have also allowed a reduction in the contrast agent dose necessary to achieve diagnostic image quality. Beyond purely morphological assessments of renal arterial wall disease, time-resolved, dynamic imaging techniques such as TRICKS, TREAT and TWIST [5] allow improved assessment of the hemodynamic significance of such abnormalities. Ultrafast gradients can likewise be utilized to perform renal perfusion imaging wherein dedicated post-processing algorithms can utilize signal intensity changes over time to calculate functional parameters such as the blood flow and tubular filtration. An overview of the impact of field strength, parallel imaging, and dynamic imaging techniques as well as the use of improved gadolinium chelates on renal MRA will be provided in this review.

## Optimizing MRA-Sequences at 1.5 and 3 T

**Figure 1:**
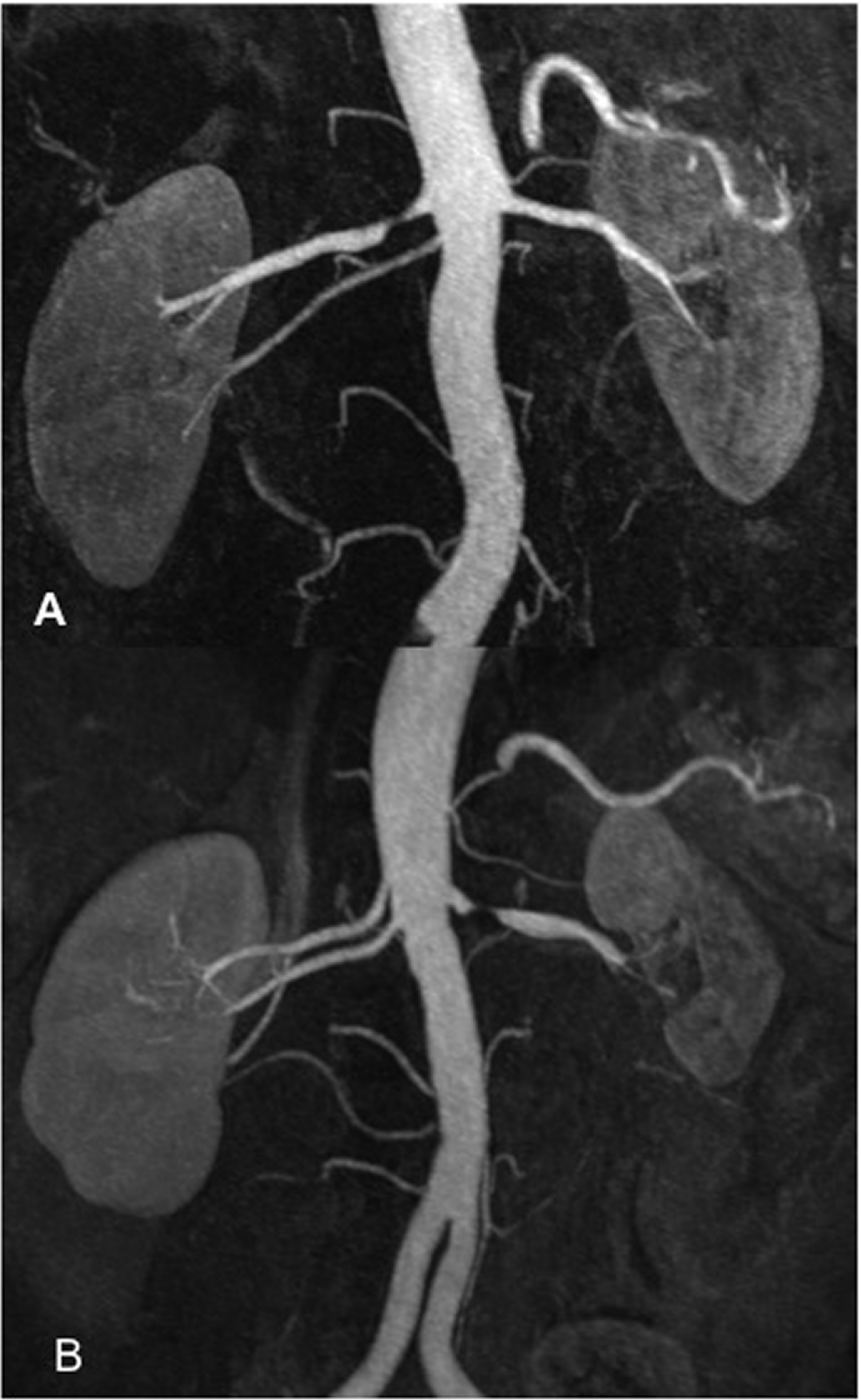
**Two thin MIP reconstructions of MRA scans from two different patients (A, B) are presented**. The high-grade renal artery stenoses are well-depicted on both scans. In the second patient (B), the renal artery stenosis has led to a renal infarction with resultant parenchymal scarring and shrinking of the entire kidney.

**Figure 2:**
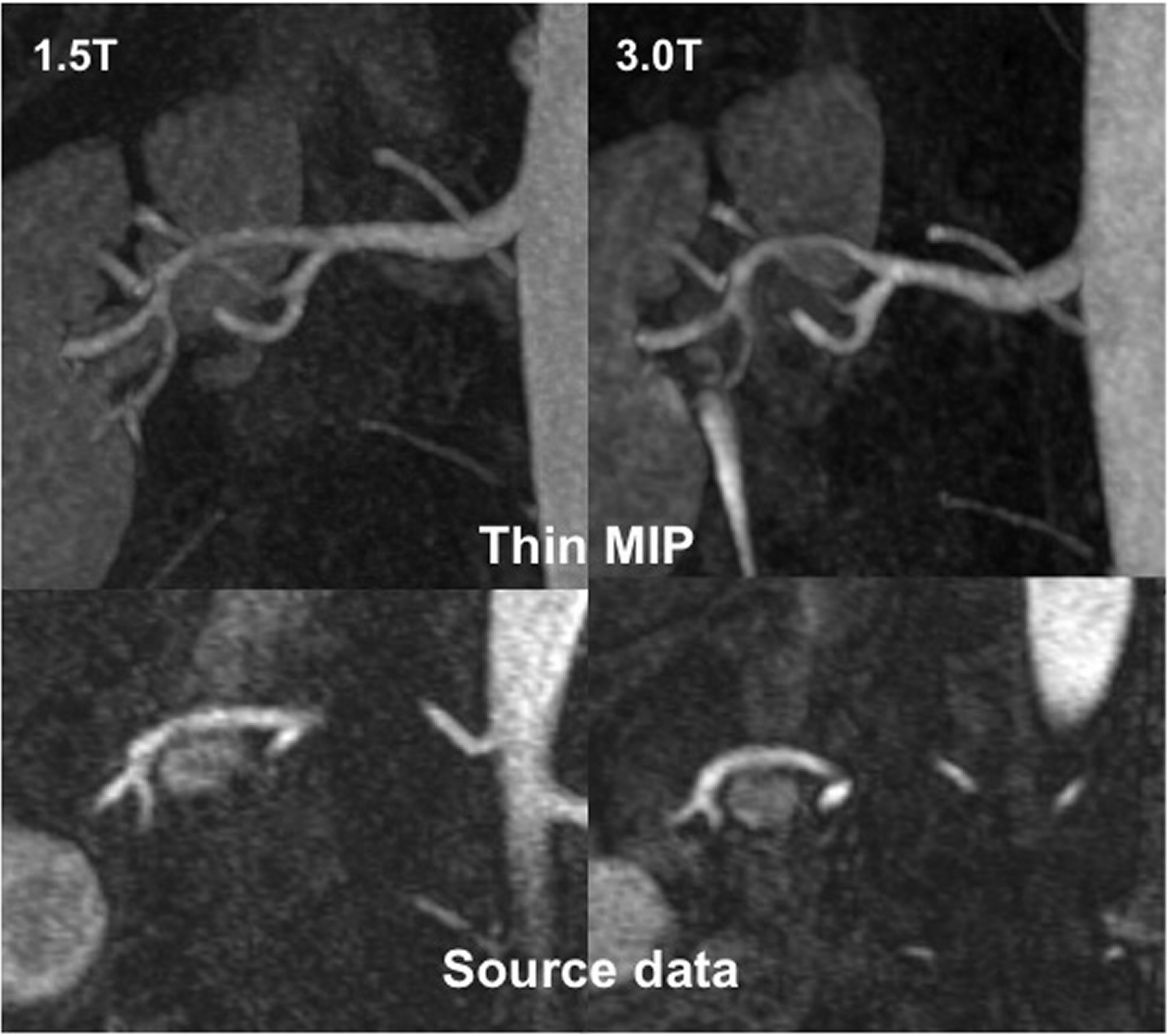
**Source data as well as thin MIP reconstructions of renal MRAs are provided, acquired in the same patient at 1.5 and 3 T**. Although the renal vasculature is clearly depicted at 1.5 T, the SNR and CNR gains at 3 T provide even more homogeneous vessel signal, improving visualization of smaller subsegmental branches.

**Figure 3:**
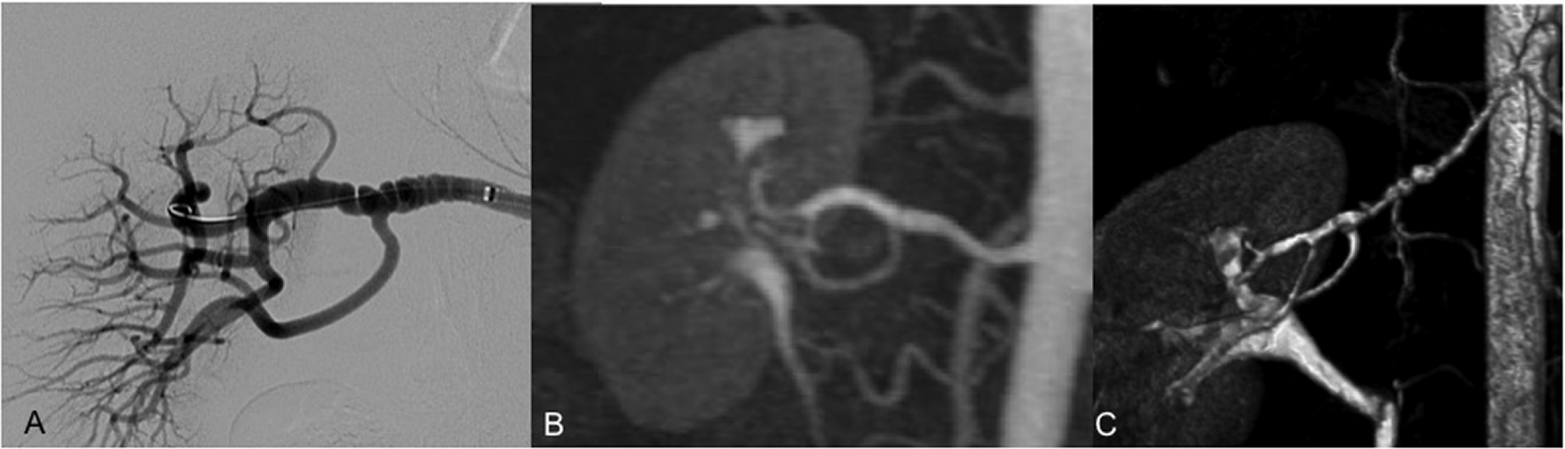
**This case illustrates the benefits of higher fields strengths for MRA: in distinction to DSA (A), on which a string-and-bed like appearance of the right renal artery consistent with fibromuscular disease is depicted clearly, this morphology is hardly visible on the corresponding MRA scan (B), acquired on a 1.5 T scanner**. Thin MIP reconstructions of a renal MRA, performed on a 3 T scanner in a different patient (C), demonstrate similar vessel changes very clearly.

Independent of the body region being examined, successful MRA depends balancing requirements of temporal and spatial resolution. In particular with respect to detecting segmental renal arterial pathology, an isotropic spatial resolution with a voxel size < 1 mm^3 ^is ideal. Furthermore to accurately measure renal artery stenosis, a high spatial resolution is desirable (Figure 1). Typically an in-plane resolution of 1 mm can be achieved with current 1.5 T MR-scanners and dedicated multi-element coils (Figure 2). As noted above, isotropic voxel sizes are optimal, as they exploit the three-dimensional character of MRA-datasets, allowing for multiplanar reconstructions without loss in image quality. Recent studies investigating different approaches for measuring renal artery stenosis (i.e. diameter reduction versus area reduction) have shown that the use of area measurements, achievable with isotropic voxel sizes, is far more accurate [4]. The depiction of subtle renal artery changes, as in FMD, which can be particularly difficult to detect if distal in location, is facilitated when high-spatial resolution MRA is also obtained with a short acquisition time [6] (Figure 3).

Voxel size and SNR are inversely related, meaning that keeping all other factors equal, increasing spatial resolution always reduces SNR and thus potentially overall image quality. Reductions in scan acquisition time can likewise deteriorate image quality. So how can MRA protocols be optimized when high-spatial resolution, short acquisition times, and optimal image quality are all desired? Although there is no perfect solution to this dilemma, parallel imaging, greater field strengths, and dedicated contrast agents are helpful in this regard.

In parallel imaging, the inherent ability of coil elements in multi-element coils to localize MR signal along the phase encoding direction is utilized to reduce the number of phase-encoding steps--a major factor influencing scan acquisition time. Such under sampling can be employed to acquire images with the same spatial resolution at a shorter acquisition time or alternatively to acquire higher spatial resolution images at the same acquisition time. The degree of under sampling utilized is reflected by the parallel imaging acceleration factor. Various vendor specific parallel imaging reconstruction algorithms are available working either in the image domain such as SENSE (Sensitivity Encoded) [7] or the k-space domain such as GRAPPA (Generalized Auto-calibrating Partially Parallel Acquisition Technique) and SMASH (Simultaneous Acquisition of Spatial Harmonics) [8]. While each algorithm allows under sampling, the techniques possess different properties. SENSE acquires so-called reference lines before the actual acquisition, which renders it susceptible to patient motion occurring between these two steps. With GRAPPA, reference lines are integrated into the acquisition rendering it less motion sensitive. The major drawback with GRAPPA is that this type of integration results in a smaller effective acceleration factor. Regardless of the technique utilized, the value of parallel imaging for abdominal MRA has been proven in various studies [9-11]. Specifically, Born et al demonstrated that SENSE parallel imaging significantly improved scan quality and reduced the necessary patient breath-hold time [11]. The acquisition time in that study was also shorter and spatial resolution higher with parallel imaging. The improved spatial resolution enabled improved depiction of the distal renal arteries; although, these improvements came at a slight, but measurable SNR loss. As these studies were conducted at 1.5 T, the acceleration factors used were 2 and 3. Clinically, acceleration factors of at least 2 are preferred. Although parallel imaging acceleration is most typically employed in the phase-encoding direction, implementation of advanced coil systems, such as high density coils, allows under sampling in both the phase and partition-encoding directions [12]. The shorter scan acquisition times facilitated by parallel imaging allow depiction of the distal renal arteries and greatly improve the robustness of MRA in critically ill patients. Moreover, the probability of diaphragmatic motion artifact degrading scan quality, which can occur in younger patients as well, is also reduced [13].

Imaging at higher field strengths, such as 3 T, is one way to address the decreases in SNR associated with higher parallel imaging acceleration factors. SNR is theoretically at least doubled at 3 T compared to 1.5 T [14]. Studies investigating renal MRA at 3 T have thus utilized acceleration factors of 3 and greater [14-17]. Due to the progressive installation of 3 T scanners worldwide, the SNR gains of higher field strengths have become more widely available. Up to 20% of the newly installed MR-scanners currently operate at 3 T. The simplest way to appreciate the gains possible with 3 T MR is to increase the readout bandwidth, hence reducing the acquisition time. The greater SNR also allows increases in spatial resolution to the sub-millimeter level with similar image quality compared to standard 1.5 T imaging [14]. To maintain reasonable acquisition times in high-spatial resolution 3 T imaging, implementation of parallel imaging is essential.

The benefits of performing MRA on 3 T systems extend beyond the theoretical doubling of SNR. Due to the prolonged T1 relaxation times of the stationary background tissue at 3 T, differences in signal between the contrast-enhanced vessel lumen and the stationary background tissue are accentuated. Smaller and more peripheral vessels are consequently depicted more clearly. This feature of 3 T MRA enables contrast agent dose reduction. However, the implementation of 3 T MR imaging is not without new dilemmas. One major problem is the four-fold increase in SAR (specific absorption rate)--a factor often strictly limited by local or national safety regulations. There are three principle ways to address SAR concerns, but all warrant careful consideration due to potentially detrimental effects on image quality. Repetition times can be increased, but at the expense of scan acquisition time. The flip angle can also be reduced; although, this may negatively affect T1 image contrast. Utilization of parallel imaging is the third means of obviating the SAR problem. Implementation of parallel imaging results in a decreased number of RF-pulses and thus diminished energy deposition.

Typical protocols for a state-of-the art renal MRA at 1.5 T and 3 T are given in Table 1.

**Table 1:** Specific sequence parameters for the MRA at 1.5 T (Siemens Avanto) and 3.0T (Siemens TimTrio) using a 6-element body matrix coil and the inbuilt 32-element spine coil

	1.5 T MRA slow	1.5 T MRA fast	3.0 T MRA
**TR/TE [ms]**	3.77/1.39	3.77/1.39	3.14/1.1

**Flip angle [°]**	25	25	23

**Bandwidth [Hz/Px]**	350	350	510

**Matrix**	512 × 80%	512 × 80%	512 × 80%

**FOV [mm^2^]**	400 × 87%	400 × 87%	400 × 81%

**Phase Oversampling [%]**	0	0	8

**Voxel size [mm^3^]**	0.8	0.8	0.65

**Spatial resolution [mm^3^]**	1 × 0.8 × 1	1 × 0.8 × 1	0.9 × 0.8 × 0.9

**Scan time [s]**	26	19	18

**Partitions**	80	80	96

**Parallel imaging**	GRAPPA factor 2	GRAPPA factor 3	GRAPPA factor 3

### Bolus Timing Techniques

**Figure 4:**
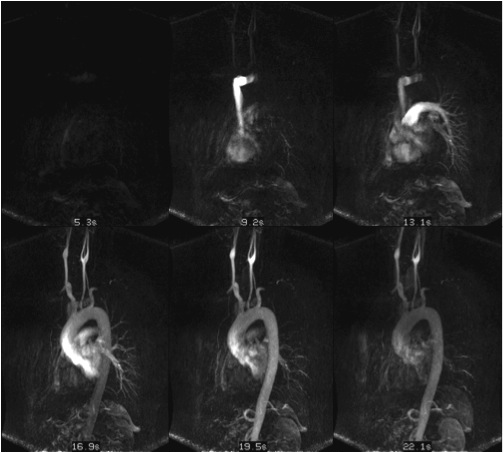
**An example of MR-fluoroscopy is illustrated**. MR-fluoroscopy can be used in place of test bolus techniques. In MR-fluoroscopy, continuous image acquisition with online image reconstruction is performed. Whenever the contrast bolus arrives in the area of interest, acquisition of the MRA-sequence can be either manually or automatically initiated. Even with the time-efficiency and ease-of-use of this technique, the abrupt switch from fluoroscopic measurements to the actual MRA-acquisition often minimizes the time for the patient to inspire for the breath hold, consequently increasing the risk of motion artifact.

Optimal contrast agent bolus timing is an additional prerequisite for high-quality MRA of the renal arteries. If the imaging acquisition is begun too early relative to the contrast bolus, ringing artifacts may impair image quality. If the acquisition is delayed, parenchymal and venous enhancement may likewise degrade image quality. One common method to achieve successful bolus timing is to use the test bolus technique. This is based on the injection of a small amount (typically 1 ml) of contrast agent at the same rate to be utilized for the injection of the actual bolus. To calculate the individual circulation time, a single slice at the level of the renal arteries is acquired. Subtracting the time the test bolus needs to arrive in the area of interest from the time-to-center gives the delay at which the scan should be started. MR-fluoroscopy can also be used for this purpose. This technique employs continuous image acquisition with contemporaneous image reconstruction (Figure 4). Once the contrast bolus arrives at the area of interest, the full MRA-sequence is either manually or automatically started. The fluoroscopic technique has the advantage of being more time-efficient and easier to implement than the test bolus approach. The major disadvantage of MR fluoroscopy is the short period of time between the fluoroscopic and actual MRA acquisition. This leaves the patient less time to initiate the breath hold necessary for the acquisition, thus resulting in a higher risk of diaphragmatic motion artifact.

### Time-resolved techniques

Even at sites with a great deal of experience with the technique, static MRA acquisitions can fail, most frequently as a result of poor bolus timing. As such, time-resolved imaging techniques offer an appealing alternative. Time-resolved MRA eliminates the need for a test bolus or MR fluoroscopy, as the acquisition is started upon injection of the contrast bolus. Modern time-resolved MRA based on view-sharing was first introduced by Korosec et al [5]. In this technique, called TRICKS, k-space is divided into central and peripheral portions containing contrast and image detail information, respectively. As the k-space center is updated more frequently than the periphery, which is relatively under sampled, contrast information is fully sampled at temporal resolution sufficient to fully resolve the passage of contrast bolus. Newer sequences combining view-sharing and parallel imaging are named TREAT and TWIST. The combination of parallel imaging with view-sharing allows further increases in temporal resolution. The benefits of time-resolved imaging exceed accurate stenosis detection alone: the hemodynamic significance of such changes can also be assessed. Moreover, pure arterial and venous phases are depicted. Time-resolved MRA is, of course, not without limitations. First, the tradeoff of the improved temporal resolution is a reduction in spatial resolution far below that achievable with static MRA-exams. Second, particularly for abdominal time-resolved MRA, the coordination of the sequence start and the presumed arrival of the contrast bolus at the target site with a given patient's breath hold capabilities render abdominal time-resolved MRA susceptible to motion artifacts. As noted above, low-dose time-resolved MRA is often acquired instead of a test bolus measurement prior to static MRA. For this purpose, minor motion artifacts and the relatively lower spatial resolution are of little consequence.

## Contrast agents

Any approved gadolinium-chelate MR-contrast agents (hereafter referred to as ECCM - extracellular contrast agents) can be theoretically administered for renal MRA. The diagnostic performance of these ECCM in controlled clinical trials with comparison to intra-arterial angiography is comparable [18,19]. Traditionally, renal MRA has been performed with both single (0.1 mmol/kg) and double (0.2 mmol/kg) dose ECCM. However, most studies have found a single dose to be sufficient [20,21] and to reveal less degradation from background parenchymal enhancement [22]. Despite this, double doses were still widely used clinically prior to the advent of nephrogenic systemic fibrosis (NSF), which has dramatically changed dosing of Gd-based ECCM in clinical MRA [23]. NSF describes a systemic body collagenosis, most likely evoked by the deposition of Gadolinium ions in body tissue. NSF is most commonly reported in patients with markedly impaired renal function after repeated injections of high doses of Gd-based ECCM. In addition to dose, the chemical structure of the injected chelate, which defines its molecular stability, has a major impact on the risk of developing NSF. Various studies have demonstrated reduced stability of linear, non-ionic chelates relative to their macrocyclic counterparts [24]. According to the new European Medicines Agency (EMA)-guidelines, linear ECCMs Gd-DTPA and Gadodiamide are contraindicated in patients with a eGFR < 30 ml/min [25]. For the linear Gd-BOPTA and the macrocyclic chelates gadobutrol and gadoterate meglumine, there is no such contraindication in this patient group; although, dose minimization is recommended. Currently, only the macrocyclic chelates are considered low-risk with regard to the risk of NSF. However, there remains, even with the macrocyclic agents, a strong clinical impetus to minimize contrast dose. Higher relaxivity gadolinium chelates have been shown to maximize signal intensity in abdominal and abdominopelvic imaging in several studies [26-28]. These studies also demonstrated that such improvement in vessel signal facilitates visualization of smaller vessels. The use of a single dose of contrast agent has also been fostered by the trend to acquire MRA examinations at 3 T. The results of studies at 3 T have indicated that single dose 0.5 M ECCM at 3 T yields similar diagnostic image quality despite lower SNR than double dose 0.5 M ECCM at 1.5 T [29], and that comparable image quality can be achieved with single dose 0.5 M ECCM at 3 T despite acquisition at higher spatial resolution [30]. For peripheral MRA a sub-single dose of 0.07 mmol/kg (as opposed to the typical single dose of 1 mmol/kg) has been shown sufficient to achieve homogeneous enhancement throughout the peripheral vasculature [31]. This low of a dose seems likely to be feasible for renal artery MRA as well. When utilizing sub-single doses, the enhancement properties of the contrast agents becomes more important as shown in an animal study where half dose gadobutrol was found superior to Gd-DTPA and Gd-BOPTA [32].

### NCE Techniques

**Figure 5:**
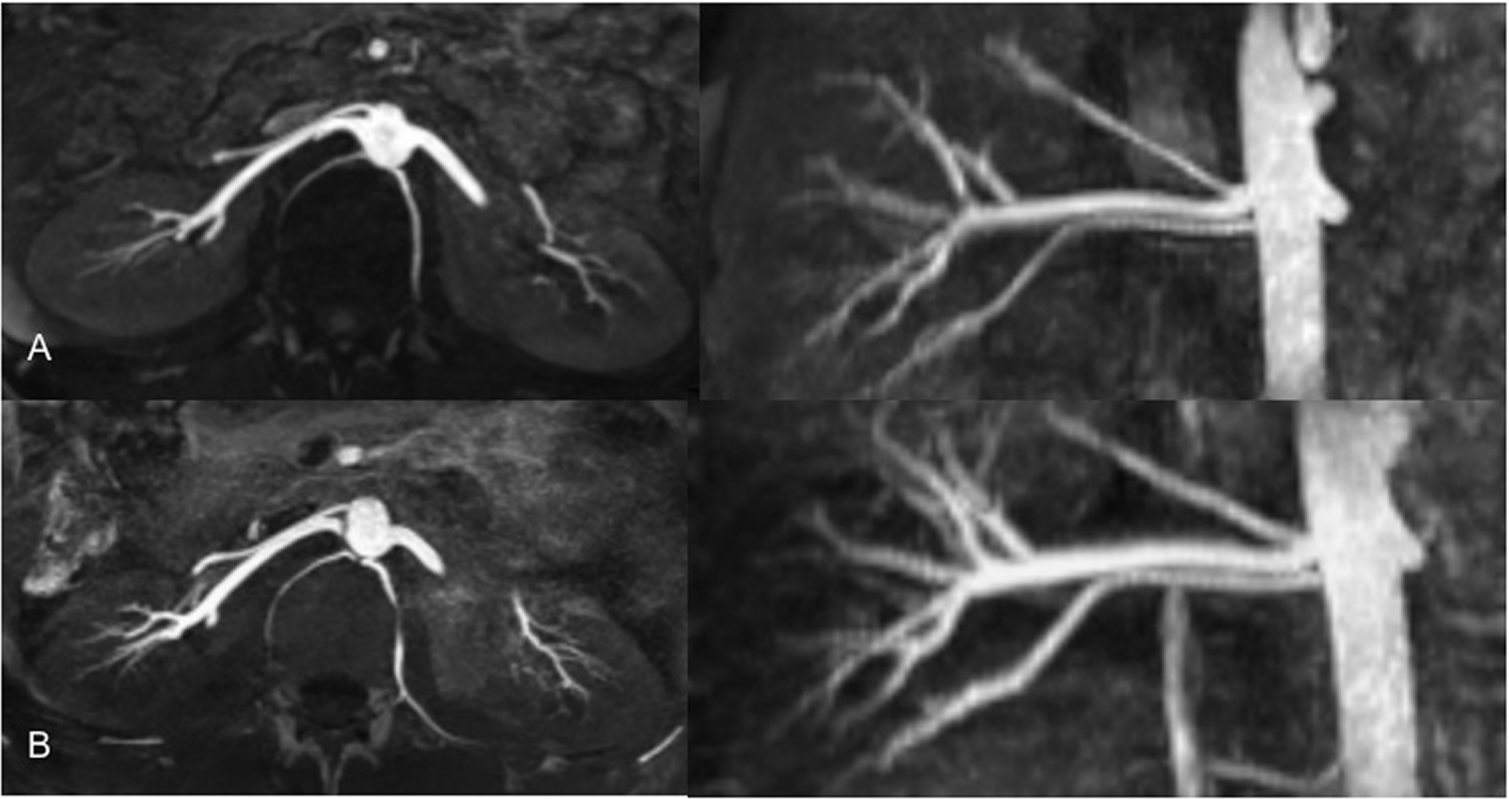
**Axial and coronal reformats of native TrueFISP renal MRAs are shown, acquired at 1.5 (A) and 3 T (B)**. These techniques are based on the inflow of arterial blood into the imaging plane, which is made visible by a SSFP-type of read-out scheme which is available in numerous vendor specific variations. Whereas, NCE-MRA appears to be a suitable screening tool for renal artery stenosis detection, accurate stenosis grading might not always be feasible. Courtesy of PD Dr. Blondin, University of Dusseldorf.

In light of the trend to decrease contrast agent dose, and because two of the most widely used macrocyclic ECCMs have long time not been approved in the United States, there has been a renewed interest in non-contrast enhanced (NCE) MRA. The newer NCE MRA techniques are based on the inflow of arterial blood into the imaging plane--a property made possible by a SSFP-type read-out scheme. Numerous vendor-specific variations on this technique are available. These differ as to whether ECG-gating is necessary, the type of respiratory gating or type navigator utilized, the primary acquisition plane, and the specific preparation pulses included [33-37]. As of now, NCE-MRA appears to be a suitable screening tool for detection of renal artery stenosis; although, accurate stenosis grading may not always be possible. As with CE-MRA, NCE-MRA benefits from the transition to 3 T where the inflow signal is stronger for SSFP-type approaches and decay of arterial labelling is slower for spin-labelling techniques (Figure 5). In patients with low cardiac output, the inflow-effect of the blood is less pronounced, and scanning such patients requires longer acquisition times so as to overcome this technical limitation. Most NCE-MRA techniques rely on a transversely oriented slab, thus rendering the technique prone to missing aberrant or accessory renal arteries. Recent NCE-MRA studies at 1.5 and 3 T in renal transplant patients with limited respective cohorts of 13 and 20 subjects have demonstrated a high diagnostic accuracy for stenosis detection with a sensitivity of 100% and a specificity of 88% relative to intra-arterial angiography [38,39]. NCE-MRA is an ideal technique by which to evaluate renal transplant grafts as the transverse imaging slab is sufficient to cover the transplant artery, and the administration of contrast material to these patients might be contraindicated depending on local guidelines regarding contrast administration.

## MRA Limitations and Areas for Improvement

**Figure 6:**
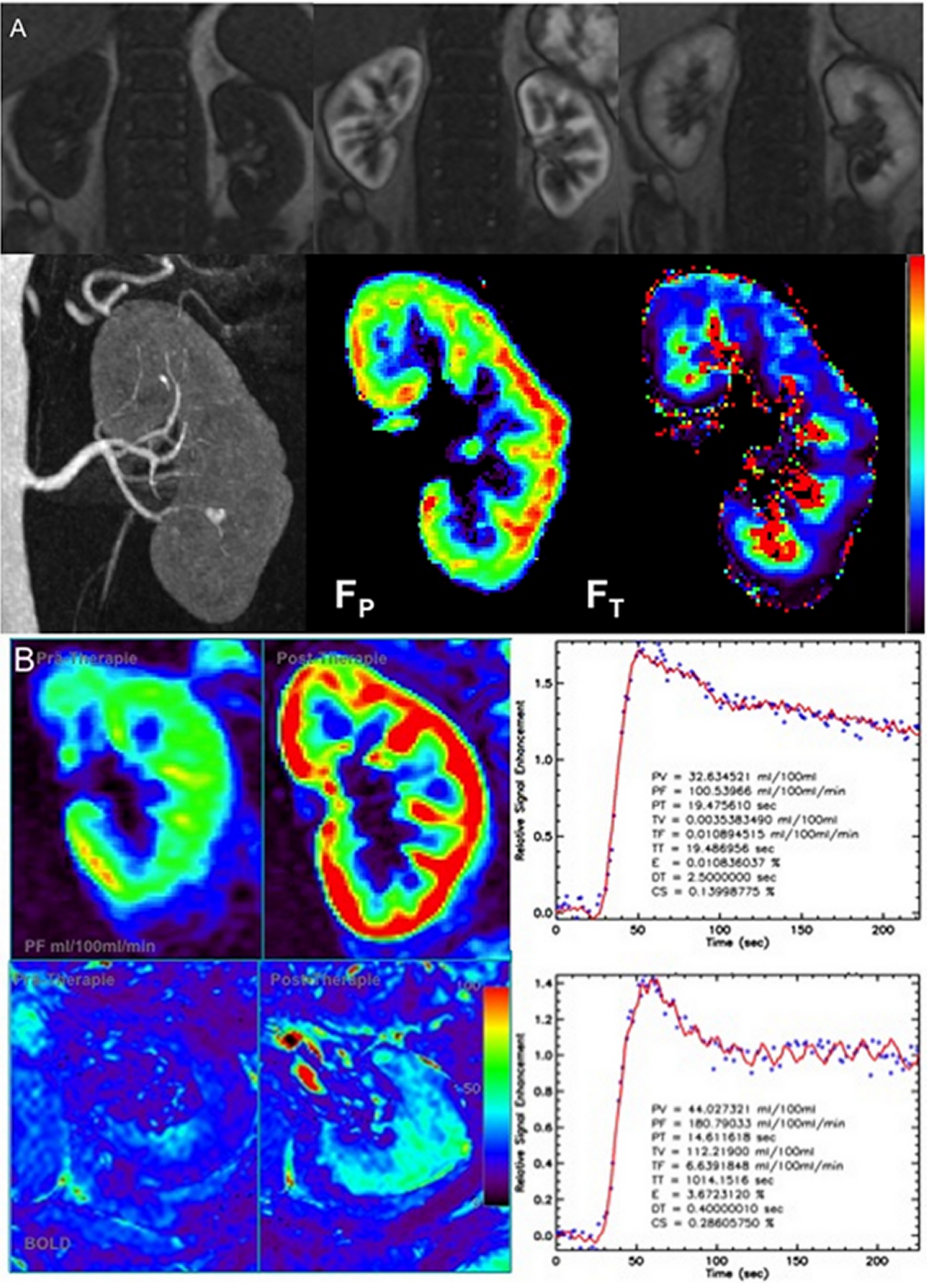
**Using dedicated post-processing, renal perfusion and filtration can be quantified based on high temporal resolution data sets**. Figure 6A illustrates in this instance non-enhanced, arterial phase and late venous phase images, subsequently acquired with a high-temporal resolution TurboFLASH sequence. After post-processing, color coded maps as well as quantified values of renal perfusion (plasma flow, F_P_) and filtration values (tubular flow, F_T_) can be derived from the primary MR data set. This analysis is, for example, especially helpful in evaluating therapeutic success after interventional procedures as shown in Figure 6B. After stenting of a high-grade renal artery stenosis, the plasma flow value normalized as did cortical and medullary oxygenation, both of which can be measured using a BOLD-sequence. These techniques along with BOLD, as illustrated in this case, have extended the utility of MR beyond purely morphologic assessments, allowing detection of functional renal impairments.

Today, MRA is considered the most cost-effective tool for the diagnosis of renovascular hypertension [40] with initial CE-MRA studies yielding specificities and sensitivities of up to 100% [41,42]. However, these initial results were probably biased by the small number of patients examined, as a larger multicenter study (RADISH-study) has failed to reproduce such high values, finding a low sensitivity (62%) for the detection of renal artery stenoses with CE-MRA and low interobserver agreement [43]. Recent large multicenter studies on interventional therapies (PTA ± stenting) for renal artery stenosis (ASTRAL and STAR) have failed to demonstrate a benefit versus medical therapy alone [44,45]. From these results, two conclusions can be drawn. First, there is clearly potential for improvement in the diagnostic accuracy of MRA, which may be accomplished through use of high-resolution techniques. A more recent study by Kramer et al evaluated a high resolution MRA sequence at 3 T finding high diagnostic accuracy for stenosis detection with respective sensitivity and specificity of 94% and 96% for detection of significant renal artery stenosis [16]. Imaging with higher spatial resolutions may overcome some of the problems encountered in the RADISH study in which a large proportion of patients with FMD were included. Second, selection of patients for interventional therapy should be based not only on the absolute degree of arterial narrowing but also upon functional MR parameters. Renal artery MRA can easily be extended to a comprehensive examination including MR-perfusion and filtration measurements [46-49] (Figure 6), diffusion-weighted and diffusion tensor imaging [50-53], T2-mapping, and blood-oxygen level dependent imaging [54-57]. Only an additional 5-10 minutes is required for the acquisition of these additional sequences, which provide further diagnostic information to better detect and stratify existing renal damage (Table 2). A recent study on MR-perfusion measurements by Attenberger et al has already demonstrated the value of this approach in triaging patients [58]. In that study, the inclusion of MR-perfusion measurements led to significantly higher detection rates of renal disease beyond that purely of renovascular origin. Other studies have shown that in renal transplants, MR-perfusion measurements are able to demonstrate global and focal perfusion changes, hence providing valuable hints at the origin of chronic renal failure [47,59]. Diffusion-weighted MR derived ADC (Apparent Diffusion Coefficient) values correlate with patient GFR [51] and are inversely proportional to the degree of stenosis present [60]. Similarly, increased fractional anisotropy (FA) values, reflecting the dependence of water motion on the renal parenchymal fibro structure, have been found in kidneys with unilateral renal artery stenosis and indicate the presence of renal fibrosis [50]. In a controlled trial in a murine model, the degree of renal fibrosis, as measured by specific myofibroblast biomarkers, has also correlated well with decreases in ADC values [61]. All of the aforementioned functional imaging modalities also benefit from the transition to 3 T, primarily due to the higher SNR but also as a result of the increased effects of susceptibility at the higher field strength [62-64]. Numerous other studies demonstrate that functional renal MR-imaging yields valuable information beyond purely morphological assessments. However, no controlled clinical trials have been conducted combining these different functional imaging tools in a large patient cohort undergoing interventional therapy. Such a study would be useful to identify specific factors that predict patient outcomes or allow improved assignments of patients to specific therapies.

**Table 2:** Functional renal imaging sequences

Sequence Type	Description
MR-perfusion and filtration measurements	Allow assessment of renal blood flow and calculation of split renal function

Diffusion-weighted imaging/Diffusion tensor imaging	ADC-values roughly correlate with renal function, FA-values can be used as marker for renal fibrosis

BOLD-Measurements	Specific R2* changes can be seen in various diseases (ATN, chronic rejection)

T2-Measurements	Experimental technique wherein T2 seems to correlate with renal function

## Future Advances

**Figure 7:**
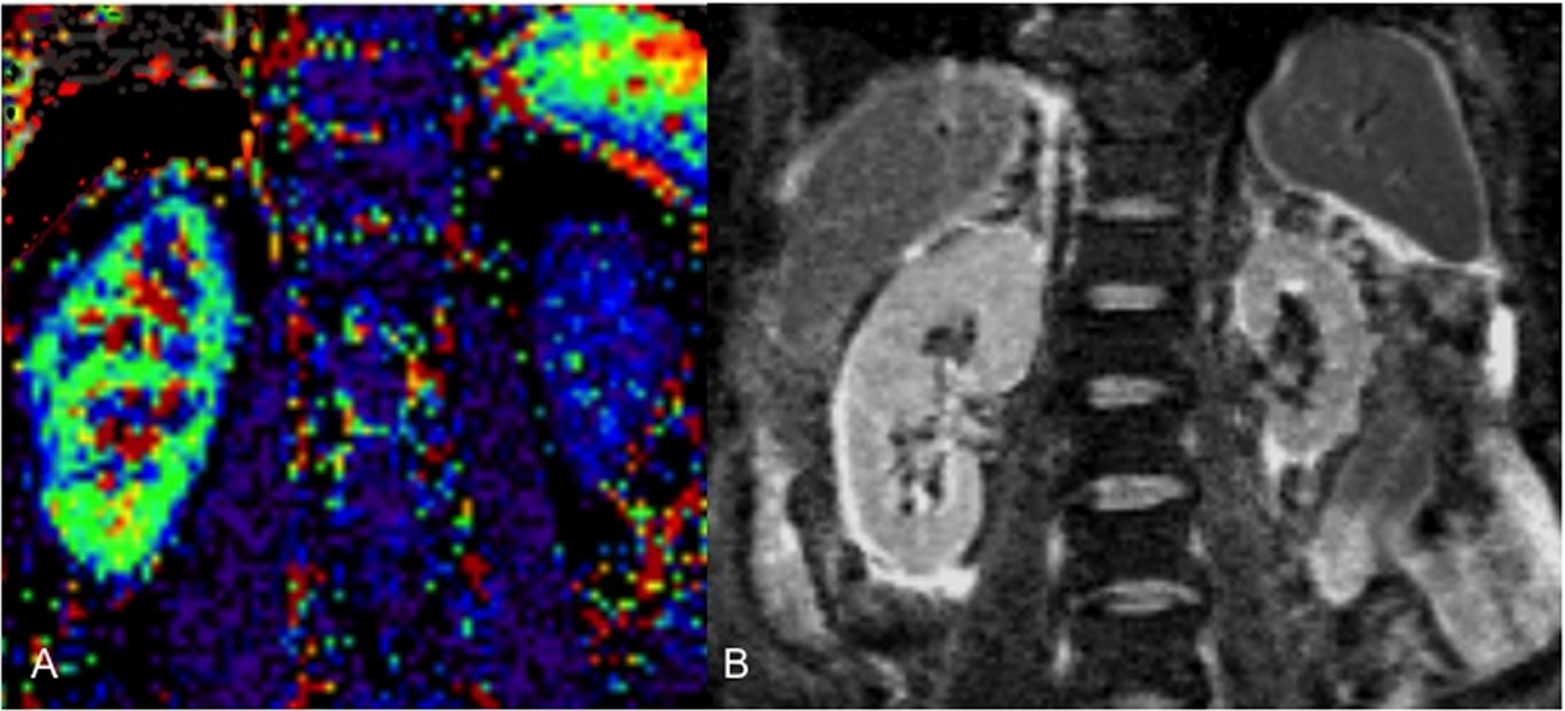
**A color coded ASL as well as an ADC map are demonstrated in a patient with a shrunken, fibrotic left kidney due to ischemia**. In this case, the ASL color coded map illustrates reduced perfusion compared to the contralateral, un-affected kidney, whereas the ADC map shows restricted diffusion. Arterial spin labeling measurements of renal perfusion have not yet been widely accepted into routine clinical practice. The principle dilemma with ASL-techniques is the inherently low signal intensity. The technique is also prone to susceptibility artifacts. Courtesy of PD Dr. Blondin, University of Dusseldorf.

What further improvements of renal MRA can we expect in the future? Arterial spin labeling measurements of renal perfusion were introduced over a decade ago [65] but have not yet found their way into broad clinical use (Figure 7). Several studies on sequence improvement and the transition to higher field strengths--the latter from which the tagging-based spin labelling techniques benefit due to the longer T1-times--have been published since that time [66]. In a clinical study on the value of ASL-perfusion imaging, Michaely et al [67] found utilization of renal ASL-perfusion measurements in combination with MR-flow measurements to distinguish healthy from diseased kidneys with specificity and sensitivity of 99% and 69%, respectively. Fenchel and colleagues applied FAIR-ASL measurements to patients with renal artery stenosis where they showed a good correlation of ASL-perfusion images with the degree of stenosis (r = 0.76) and with single photon emission tomography perfusion data (r = 0.83) [68]. The main problem with ASL-techniques is the inherent low signal intensity and its susceptibility to artifacts. Their greater clinical availability, trends toward functional renal MR assessments, and the need to minimize or stop contrast agent administration will likely foster broader acceptance of renal ASL-measurements.

**Figure 8:**
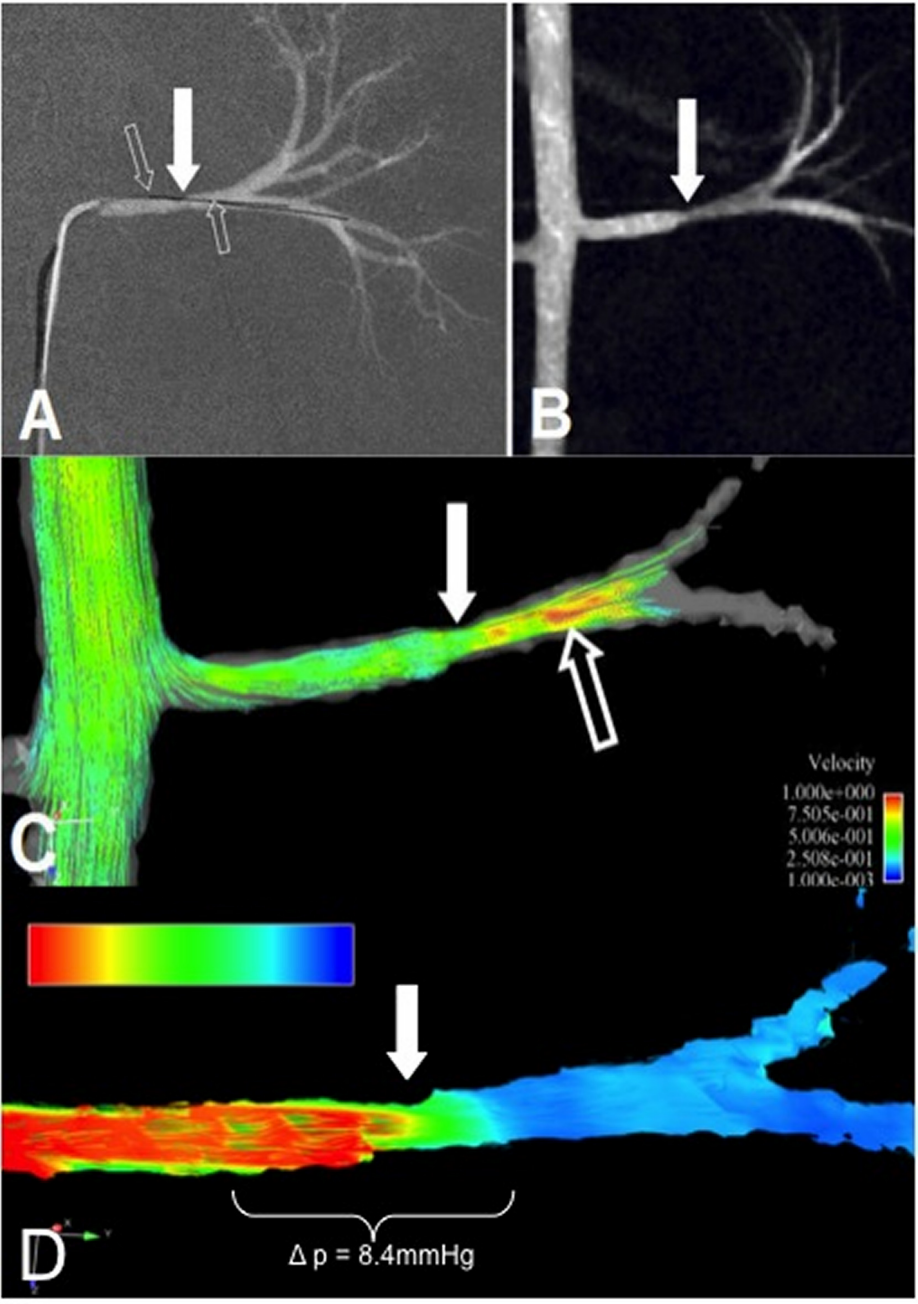
**Based on conventional phase-contrast MRA and driven by the increase in available post-processing computing power, a new vascular imaging technique with great clinical potential has evolved in the past few years: a flow-sensitive, 3-dimensional, and 3-directional time-resolved gradient echo sequence--often referred to as 7D-MRA**. This figure illustrates the evaluation of renal artery stenosis using DSA for reference (A) and MRA (B). The flow alterations around the stenosis present can not only be visualized on color coded post-processed 7D-MRA parameter maps (C) with red indicating increased post stenotic flow, but also the trans-stenotic pressure gradient can also be quantified (D) using the Bernoulli equation. Courtesy of PD Dr. Bley, UKE Hamburg.

**Figure 9:**
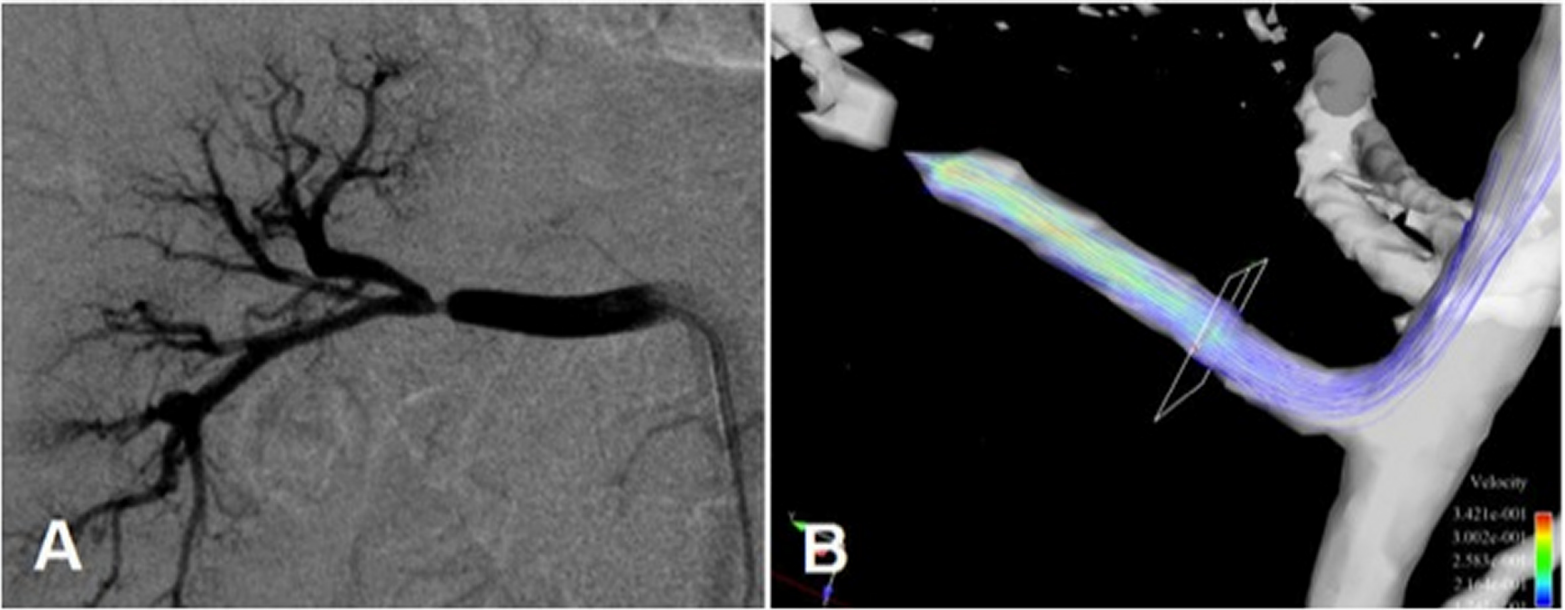
**This figure illustrates one of the disadvantages of the 7D flow technique: if the trans-stenotic pressure gradient (TSPG) exceeds > 60 mmHg, as is the case in severe, high-grade stenoses, there is often no signal distal to the severe stenosis detected, rendering measurements of TSPG impossible**. Courtesy of PD Dr. Bley, UKE Hamburg.

Based on conventional phase-contrast MRA-techniques and driven by increased post-processing computing power, a new vascular imaging technique with great clinical potential has evolved in the past years: a flow-sensitive, 3-dimensional, and 3-directional time-resolved gradient echo sequence--often referred to as 7D-MRA. Once acquired, data from 7D-MRA are analysed to yield phase contrast MR angiography, color-coded streamlines, and particle trace 3D visualization. The latter allows graphical depiction of flow patterns in a vessel. Initial results have demonstrated potential for detecting altered flow patterns in aneurysms or in patients with aortic disease [69,70]. Impressively, the technique has also demonstrated retrograde aortic arch flow in stroke patients secondary to retrograde emboli [71]. No robust data is available for this technique at present for evaluation of the renal vasculature. Based on personal communication, initial 7D-MRA data in a swine model of renal artery stenosis allows for visualization and quantification of stenotic flow acceleration (Figure 8, additional file 1 and 2). Utilizing the Bernoulli equation, trans-stenotic pressure gradients can reliably derived from the 7D-MRA data, most likely rendering this technique a pivotal tool for future interventional decision making. One minor current dilemma is the potential for the failure of particle trace calculations with high grade stenoses (Figure 9). Exemplary movies demonstrating the 7D-MRA technique can be downloaded online.

## Summary

Renal MRA is a continuing success story. Current state of the art 3 T MRA allows reliable detection of even the most subtle renal artery changes with utilization of relatively small contrast agent doses. Whether non-contrast enhanced MRA techniques continue to flourish depends upon their further development and demonstration of their clinical utility, the latter being shown thus far only in the assessment of renal transplants. In the future, MRA--whether performed with or without contrast agents--will most likely be combined with functional MR renal imaging techniques such as MR-perfusion measurements which currently lie on the brink of broad clinical application.

## Competing interests

Henrik Michaely is a consultant to Bayer. The other authors declare that they have no competing interests.

## Authors' contributions

Literature research was done by UA and HM. UA, JM, SOS and HM drafted the manuscript and approved the final manuscript.

## Supplementary Material

Additional file 1 **7D-MRA renal arteries**. Particle-trace depiction of a 7D-MRA in a swine model. The flow velocity of the blood in the renal artery can be measured. Courtesy of PD Dr. Bley, UKE Hamburg. Click here for file

Additional file 2 **7D-MRA renal artery stenosis**. The particle-trace depiction of a renal artery stenosis in a swine model demonstrates increased flow velocity at the site of the stenosis. Courtesy of PD Dr. Bley, UKE Hamburg. Click here for file
